# Draft Genome Sequences of Enteropathogenic *Escherichia coli* O103 Strains Isolated from Feces of Feedlot Cattle

**DOI:** 10.1128/genomeA.00387-17

**Published:** 2017-05-25

**Authors:** Lance W. Noll, Jay N. Worley, Xun Yang, Pragathi B. Shridhar, Jianfa Bai, Jianghong Meng, Doina Caragea, T. G. Nagaraja

**Affiliations:** a Department of Diagnostic Medicine/Pathobiology, Kansas State University, Manhattan, Kansas, USA; b Veterinary Diagnostic Laboratory, Kansas State University, Manhattan, Kansas, USA; c Department of Computing and Information Sciences, Kansas State University, Manhattan, Kansas, USA; d Department of Nutrition and Food Science, University of Maryland, College Park, Maryland, USA

## Abstract

Enteropathogenic *Escherichia coli* (EPEC) pathotype represents a minor proportion of *E. coli* O103 strains shed in the feces of feedlot cattle. The draft genome sequences of 13 strains of EPEC O103 are reported here. The availability of the genome sequences will help in the assessment of genetic diversity and virulence potential of bovine EPEC O103.

## GENOME ANNOUNCEMENT

Cattle serve as a reservoir for enteropathogenic *Escherichia coli* (EPEC), including the EPEC O103 serogroup (1). The organisms are harbored in the hindgut of cattle and shed in the feces. Human infection is mostly associated with children and can occur following fecal-oral transmission of EPEC through direct contact or via contaminated food or water (2). Although considered a major diarrheagenic pathogen in developing countries (3), EPEC infections are not common in the United States; however, outbreaks have been reported (4, 5). Unlike enterohemorrhagic *E. coli* (EHEC), EPEC strains do not carry Shiga toxin genes (*stx*_1_ and/or *stx*_2_), yet they share many important virulence genes with EHEC (6). Similar to EHEC, EPEC strains are positive for the locus of enterocyte and effacement (LEE)-carried intimin gene (*eae*), which together with other LEE-carried genes can cause microvillus destruction during human infection, resulting in the characteristic attaching and effacing lesions (7). Strains within the EPEC pathotype are further characterized as typical or atypical, depending on presence or absence, respectively, of the EPEC adherence factor plasmid (8). Although EHEC O103 has been genetically characterized (9, 10), less is known about EPEC O103. Söderlund et al. (11) published the genomes of nine EPEC O103 strains isolated from feces of cattle in Sweden, which to our knowledge, remain the only publicly available sequences to date.

Here, we report the whole-genome draft sequences of 13 strains of atypical EPEC O103 (12 O103:H2 strains and 1 O103:H11 strain) isolated from the feces of feedlot cattle in the United States (1). The DNeasy blood and tissue kit with the QIAcube robotic workstation (Qiagen, Germantown, MD) were used to extract DNA from overnight cultures of each bacterial strain. Genomes were sequenced using an Illumina MiSeq platform (Illumina, San Diego, CA), and genome libraries were constructed using the Nextera XT DNA library preparation kit and MiSeq reagent kits version 2 (500 cycles) (Illumina, Inc.). *De novo* genome assembly was performed using SPAdes version 3.6.0 (12). The accession numbers for each strain identification (ID), along with the genome size and number of contigs per genome, are summarized in Table 1.

**TABLE 1:** Characteristics of enteropathogenic *Escherichia coli* O103 strains isolated from feces of feedlot cattle

Strain	Serotype	Genome size (bp)	No. of contigs	Accession no.
UMDKSU-2013-3-73A	O103:H2	5,165,309	150	MVKZ00000000
UMDKSU-2013-3-98B	O103:H2	5,282,670	258	MVLA00000000
UMDKSU-2013-3-109D	O103:H2	5,333,739	268	MVLB00000000
UMDKSU-2013-3-141E	O103:H2	5,254,213	218	MVLC00000000
UMDKSU-2013-3-134A	O103:H2	5,187,748	199	MVLD00000000
UMDKSU-2013-3-435B	O103:H2	5,211,713	220	MVLE00000000
UMDKSU-2013-3-492A	O103:H11	5,674,533	406	MVLF00000000
UMDKSU-2013-3-296D	O103:H2	5,155,875	137	MVLG00000000
UMDKSU-2013-3-331C	O103:H2	5,239,618	191	MVLH00000000
UMDKSU-2013-3-416C	O103:H2	5,188,193	176	MVLI00000000
UMDKSU-2013-3-533B	O103:H2	5,210,719	212	MVLJ00000000
UMDKSU-2013-3-536F	O103:H2	5,205,112	200	MVLK00000000
UMDKSU-2013-3-526E	O103:H2	5,235,857	218	MVLL00000000

The publication of the sequences of these 13 strains will contribute to the currently limited amount of publicly available sequence data on EPEC O103. These genomes will allow for investigations into the genetic similarities and differences of EPEC O103 strains to other major *E. coli* pathotype-serogroup combinations, including EHEC O103. They will also serve as a valuable resource toward the study of EPEC O103 genome evolution.

### Accession number(s).

This whole-genome shotgun project has been deposited at DDBJ/ENA/GenBank under the accession numbers listed in Table 1.
